# Prospective acceptability of a multipurpose technology (MPT) implant in preclinical development to prevent HIV and unplanned pregnancy: Qualitative insights from women end users and health care providers in South Africa and Zimbabwe

**DOI:** 10.1371/journal.pone.0285711

**Published:** 2023-05-17

**Authors:** Sikhanyisiwe Nkomo, Wanzirai Makoni, Mary Kate Shapley-Quinn, Ellen Luecke, Enough Mbatsane, Kgahlisho Manenzhe, Khatija Ahmed, Leah M. Johnson, Imelda Mahaka, Ariane van der Straten

**Affiliations:** 1 Pangaea Zimbabwe AIDS Trust, Harare, Zimbabwe; 2 Women’s Global Health Imperative, RTI International, San Jose, California, United States of America; 3 Setshaba Research Centre, Soshanguve, South Africa; 4 Faculty of Health Sciences, Department of Medical Microbiology, University of Pretoria, Pretoria, South Africa; 5 RTI International, Research Triangle Park, North Carolina, United States of America; 6 ASTRA Consulting, Kensington, California, United States of America; 7 Center for AIDS Prevention Studies, University of California, San Francisco, San Francisco, California, United States of America; Boston College School of Social Work, UNITED STATES

## Abstract

**Background:**

Given the high rates of both HIV and unintended pregnancies in sub-Saharan Africa, the SCHIELD program aims to develop a multipurpose technology implant for HIV and pregnancy prevention. An end-user evaluation was undertaken with young women and health care providers to assess preferences for modifiable implant attributes to improve future adoption and rollout.

**Methods:**

Focus group discussions were conducted with potential women end users, and health care providers experienced in implant insertion or removal participated in in-depth interviews. All participants were recruited from Harare, Zimbabwe, or Soshanguve, South Africa. The purposively stratified sampled women were either implant experienced or implant naïve and were categorized into three groups: nulliparous, postpartum, or engaged in transactional sex. Topics covered included duration (six months to three years), biodegradability, removability, and independent rod retrievability (per indication). Data were analyzed using Dedoose software and summarized into emerging themes.

**Results:**

Participants identified three key areas that could facilitate rollout, uptake, and adherence of an implant for HIV and pregnancy prevention. First, discreetness was the most salient topic and was associated with implant characteristics such as anatomical location, flexibility, and biodegradability. Second, the ability to independently retrieve the HIV or pregnancy prevention component was preferred, as life circumstances may change and was favored by all participants, except for young women in Soshanguve. Third, there is a need for proper counseling, sensitization, provider training, and health campaigns to facilitate rollout of a 2-in-1 implant.

**Conclusions:**

A 2-in-1 implant was seen as highly desirable by most young women and health care providers. Participants discussed potential concerns and barriers to uptake of a biodegradable implant with dual HIV prevention and contraceptive properties, identifying key implant attributes that product developers can modify while still in preclinical stages.

## Introduction

HIV prevalence remains high in sub-Saharan Africa overall and specifically among people of reproductive-age (15 to 49 years old), and is estimated at 19% in South Africa and 13% in Zimbabwe [1]. Despite multifaceted efforts to introduce oral pre-exposure prophylaxis (PrEP) as a new HIV prevention tool, adherence to daily pill taking remains a key barrier [2]. This highlights the need for additional HIV prevention tools, including longer-acting and lower-burden methods [3, 4]. Estimated unmet need for family planning among sexually active unmarried women is 24% in South Africa [5] and 20% in Zimbabwe [6]. Given the overlapping unmet needs of both HIV prevention and contraception among young women in sub-Saharan Africa, developing new effective multipurpose technologies (MPTs) aligned with women’s preferences and their life context is a priority. Recent end-user research has shown that, in general, women prefer MPT products over single-indication methods for HIV and pregnancy prevention [7, 8]. Consequently, researchers are developing new MPTs that can simultaneously provide both HIV prevention and contraception.

Implants combining long-acting HIV and pregnancy prevention offer multiple advantages, including long duration of protection, discreetness, low opportunity for user error, and infrequent clinical visits [9]. Nonetheless, contraceptive implants have played varied roles in national family planning programs. For example, the contraceptive implant has been around for decades in Zimbabwe, whereas it was introduced in South Africa in 2014 [10]. As a result, current contraceptive implant prevalence is much higher in Zimbabwe (9% to 14%) [6] compared with South Africa (3% to 4%) [11].

Progress made with contraceptive implants has supported recent efforts toward developing implants for long-acting HIV PrEP. Diverse implant designs are under development that have advanced to preclinical or clinical testing [12]. Recently, the first Phase I human trial of implants for HIV PrEP have been conducted and demonstrated drug release in the target range for three months [13, 14]. The Subcutaneous Contraceptive and HIV Implant Engineered for Long-Acting Delivery (SCHIELD) implant is an MPT at the preclinical stage. Specific attributes of SCHIELD include biodegradable encasing polymer, one or two rods that can be subcutaneously inserted with commercially available trocars, and release of an antiretroviral and a contraception drug at a controlled rate for continuous protection from HIV and pregnancy for approximately one year. A dissolvable (or biodegradable) implant would offer the advantage of long-acting delivery without surgical removal after drug depletion. Nevertheless, the implant can be removed during the drug delivery phase in the case of an adverse event or a desired return to fertility [15].

Engaging end users early during development to optimize modifiable attributes of products has been recommended to improve chances of success with future adoption [16–21]. The present study focuses on optimizing the design of an MPT implant before it advances to human trials with the objective of it being responsive to potential end-users’ and health care providers’ feedback. We sought preferences and perspectives of diverse women between the ages of 18 and 30 as potential end users, and health care providers in South Africa and Zimbabwe with experience in the insertion and removal of implants. Feedback from end users and heath care providers will be used to inform the design of the SCHIELD implant in development, reflecting collaboration between bench scientists and potential end users. This co-design effort for new MPT technology that is better suited to the context of use may also inform future messaging and implementation strategies.

## Materials and methods

### Research setting, study design, and participants

Research activities were conducted between February 2019 and September 2019 at the Setshaba Research Centre (SRC) in Soshanguve, Tshwane, South Africa, and at Pangaea Zimbabwe AIDS Trust (PZAT) in Harare, Zimbabwe. Chitungwiza is located 30 km from Zimbabwe’s capital city of Harare. Both Harare and Chitungwiza are identified as HIV “hotspots” in Zimbabwe. Soshanguve is a large and very diverse area northeast of Tshwane, in South Africa’s Gauteng province. It was developed from three preexisting townships consisting of informal settlements and established formal housing.

The SCHIELD study used focus group discussions, in-depth interviews, and a brief quantitative survey to gather feedback from potential South African and Zimbabwean women end users and health care providers. These activities were intended to elicit preferences for modifiable attributes for the SCHIELD implant and social and structural factors that may influence future uptake.

Three categories of women were recruited to participate in focus group discussions: nulliparous (n = 40), postpartum (n = 36), and women engaged in transactional sex (n = 34), stratified by implant experienced or naïve status. Recruitment strategies were dependent on the participant group, and included referrals from clinics, hospitals or health facilities, visiting youth groups, door-to-door recruitment in the community, hotspots for women engaged in transactional sex (e.g., taverns, social grants), mobilization from learning institutions, nongovernmental organizations, and faith-based organizations. Eligibility criteria included being between age 18 and 30, HIV-negative status by self-report, and fluent in one of the study languages (Shona in Zimbabwe, Tswana in South Africa, or English at either site).

In-depth interviews were conducted with health care providers who had experience administering/removing implants and other key stakeholders with influence in HIV and/or contraceptive technology implementation in South Africa and Zimbabwe, such as community health workers and reproductive health supervisors or officials. Health care providers were recruited from health care facilities such as public clinics, private clinics, nongovernmental organizations, faith-based organizations, and drop-in centers.

### Data collection

During the focus groups discussions and in-depth interviews, the SCHIELD implant was introduced as an MPT currently in preclinical development. It was presented as a biodegradable product that will be subcutaneously inserted via a trocar/applicator, delivering two medicines: one for HIV prevention and one for contraception. Specifically, the women who participated in focus group discussions were informed of their role in the study as “co-designers” or “fellow scientists” who would help shape the design of SCHIELD and help decide on the final form that would advance to clinical trials.

End-user feedback was gathered on topics such as coformulation as compared with coadministration (HIV and pregnancy prevention medicines delivered in the same implant rods or separately, respectively), implant insertion considerations (e.g., anatomical insertion sites, reducing pain and scarring), implant design characteristics (e.g., biodegradability, retrievability), applicator characteristics (e.g., previous experiences with trocar devices, single as compared with reusable systems), and perceived social adoption factors for end users (e.g., community education).

**Fig 1:**
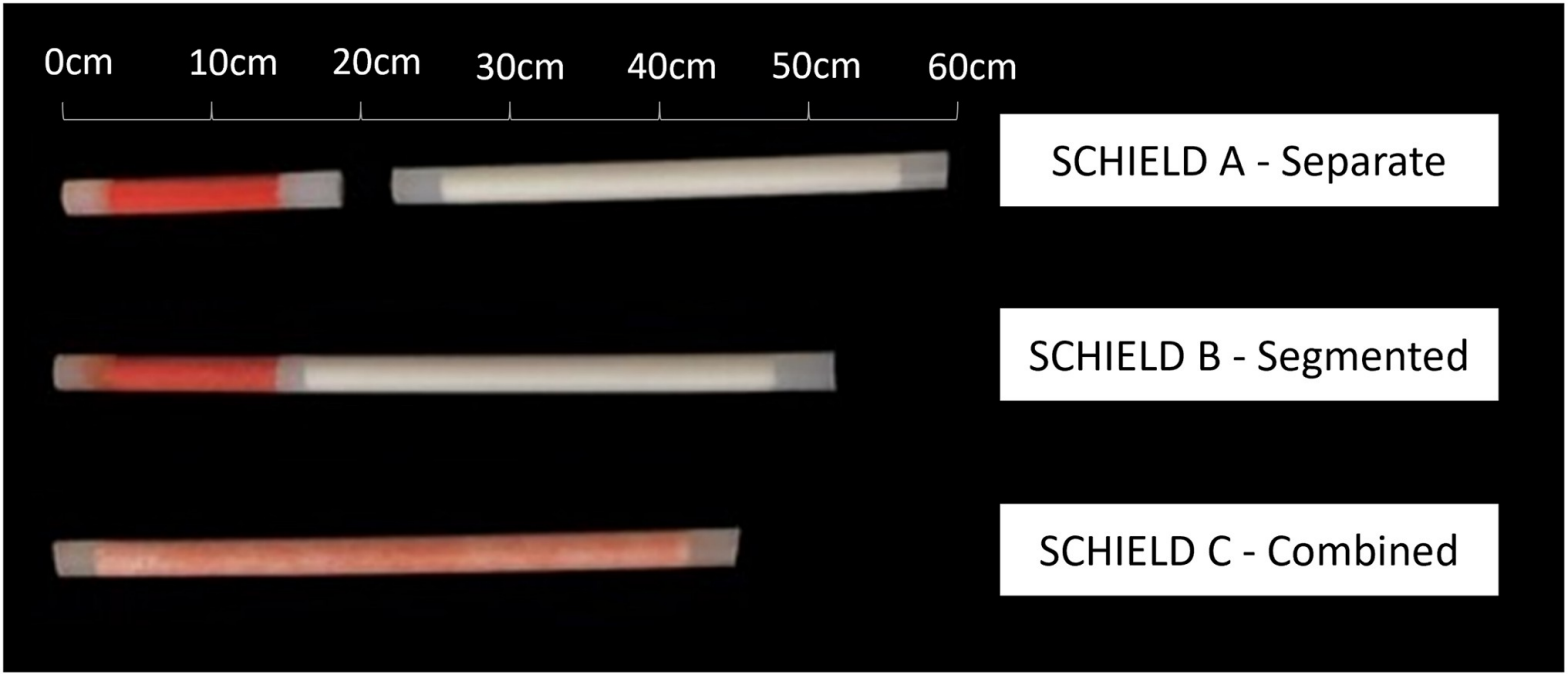
SCHIELD prototypes shown to all participants. Color difference accentuated for illustrative purposes and not identical to prototypes viewed by participants. Orange indicates the contraceptive component and white indicates the HIV prevention component.

To facilitate data collection, all participants had an opportunity to look at different SCHIELD implant prototypes, contraceptive implants, and photographs of trocars/applicator systems. This helped highlight what features end users identify as most influential to their willingness to use the implant system in the future. Participants were invited to hold and touch the prototypes (which were placed in sealed plastic bags) and two existing products Implanon (etonogestrel implant) and Jadelle (levonorgestrel implant). This enabled participants to compare attributes such as flexibility and length between existing contraceptive implants and the SCHIELD prototypes. Three SCHIELD prototypes were shown to participants: SCHIELD A (“separate”) with one long rod and one short rod; SCHIELD B (“segmented”) with both medicines in different compartments of one rod; and SCHIELD C (“combined”) with both medicines combined throughout a single rod, as shown in Fig 1.

In the in-depth interviews, health care providers also handled and discussed the implant prototypes. In addition to the exploration of the same topics as in the end-user focus group discussions, health care providers provided feedback on trocar/applicator characteristics (e.g., previous experiences with trocar devices, single as compared with reusable systems), and service delivery capacity (e.g., current training processes for administering implants, capacity of health care system to provide products).

All focus group discussions and in-depth interviews were conducted by trained social scientists in either in English, Shona, or Setswana dialect per the participant’s preference. Interviews lasted approximately one hour and focus groups lasted approximately 2 hours. Focus groups and interviews were audio-recorded (with consent), transcribed, and translated by interviewers, undergoing quality checks at the study site and by the US-based data center, RTI International’s Women’s Global Health Imperative. Interviewers also completed debrief reports after completion of the focus groups or interviews to summarize participants’ key responses and general flow of the sessions. Before participating in a focus group or interview, all participants completed a short interviewer-administered quantitative survey that collected demographic information. Key data on implant preferences also were collected through a survey after completing a focus group or interview.

### Data analysis

Demographic and preference data were analyzed descriptively using Stata version 16.1. Verbatim transcripts were coded in Dedoose version 8.2.18. The qualitative codebook was developed collaboratively by the coding and analysis team. The team met regularly to reflect on the textual data, discuss any text where code applications were unclear or difficult, and resolve coding differences. Intercoder reliability was assessed through these conversations and by having one coding team member whose primary role was to review all team member’s coding on all transcripts (through full transcript review at first, and later transitioning to spot checking once reliability was established through discussions). This team member raised any issues with inconsistencies, any differences in code application were discussed, and resolved through consensus. Codebook updates were made, as necessary, to reflect the team’s decision.

For this article, the team generated code reports by pulling data coded as “Accessibility,” “Barriers,” “Enablers,” “Retrievability,” “Number of rods,” “Discreetness,” and “Biodegradability.” These codes were selected to provide key insights for product developers into contextual issues and product features that could impact future use of an MPT implant. Based on these code reports, the team completed analytical memoranda to summarize key themes and findings. Interpretation and summarization of the most salient findings were discussed by the analytical team to reach consensus.

### Ethical approval

The study protocol was reviewed and approved by each data collection site’s respective institutional review boards: The Medical Research Council of Zimbabwe (MRCZ) and the Research Council of Zimbabwe (RCZ) in Harare, Zimbabwe; and Pharma Ethics Committee (PEC) in Soshanguve, South Africa. Written informed consent was obtained from all participants prior to data collection procedures and all participants were reimbursed for their time and transportation costs in accordance with local IRB requirements.

## Results

### Participant characteristics

In total, 13 focus group discussions were conducted with 110 young women in South Africa and Zimbabwe, and 17 in-depth interviews were conducted with health care providers who had prior experience with inserting and/or removing contraceptive implants. Of the 110 focus group participants, more were unmarried at the South African site than the Zimbabwean site, reflecting the differing norms for marriage of young women in the study communities. There also were notable differences in self-employment (more common at the Zimbabwean site) and HIV and pregnancy prevention methods ever used. Prior use of male condoms and injectables as contraceptive methods were more frequently reported by South African women than Zimbabwean women, and more Zimbabwean women reported never having used any contraceptive method. Further demographic characteristics of focus group participants are described in Table 1.

**Table 1:** Demographic characteristics of all focus group discussion participants, by site and overall.

	South Africa (SRC) (n = 46)	Zimbabwe (PZAT) (n = 64)	TOTAL (n = 110)
**Age mean (median, min-max)**	24.4 (23.5, 18–34)	23.3 (23, 18–30)	23.8 (23, 18–34)
**Unmarried** *	44 (96%)	48 (75%)	92 (84%)
**Nulliparous**	18 (39%)	29 (45%)	47 (43%)
**Ever engaged in transactional sex** *	27 (59%)	22 (34%)	49 (45%)
**Level of education**			
Primary	0 (0%)	4 (6%)	4 (4%)
Secondary, not complete	11 (24%)	15 (23%)	26 (24%)
Secondary, complete	27 (59%)	36 (56%)	63 (57%)
College/university	8 (17%)	9 (14%)	17 (16%)
**Source of income** *			
None	29 (63%)	22 (34%)	51 (46%)
Student	10 (22%)	11 (17%)	21 (19%)
Self-employment	1 (2%)	24 (38%)	25 (23%)
Formal	6 (13%)	7 (11%)	13 (12%)
**Contraceptive/HIV prevention methods ever used**			
None	4 (9%)	14 (22%)	18 (16%)
Male condom *	34 (74%)	34 (53%)	68 (62%)
Female condom	2 (4%)	1 (2%)	3 (3%)
Vaginal gel	3 (7%)	6 (9%)	9 (8%)
Pills	16 (35%)	24 (38%)	40 (36%)
IUD	1 (2%)	2 (3%)	3 (3%)
Implants	17 (37%)	24 (38%)	41 (37%)
Injectable *	30 (65%)	11 (17%)	41 (37%)
Other	0 (0%)	1 (2%)	1 (1%)
**Type(s) of contraceptive implants ever used**	n = 17	n = 24	n = 41
Norplant	0 (0%)	1 (4%)	1 (2%)
Jadelle *	1 (6%)	21 (88%)	22 (54%)
Implanon *	16 (94%)	2 (8%)	18 (44%)
Don’t know	0 (0%)	0 (0%)	0 (0%)

*Note*: SRC, Setshaba Research Centre; PZAT, Pangaea Zimbabwe AIDS Trust.

* p < 0.05 using Fisher’s exact test

The measures outlined in Table 1 also were examined by implant experience status. Similar demographic characteristics were reported by both implant- experienced (n = 41) and implant-naïve (n = 69) groups, apart from age. Implant- experienced participants were on average older than participants who had not used an implant previously (25.6 as compared with 22.7 years, p = 0.0002).

A majority of the 17 health care providers who had prior experience with inserting and/or removing implants were female. Pertinent demographic and professional characteristics of these providers are presented in Table 2. They worked in a range of job settings, such as public hospitals and clinics, private clinics, clinics run by nongovernmental organizations, research clinics, and youth-friendly clinics. Health care providers in Harare alternate service provision between the public- and private- health sector. Consequently, they were able to talk about their different experiences in providing contraceptive implants between the two sectors. The longer history of availability of contraceptive implants in Zimbabwe was reflected in the providers’ length of experience, with those in Zimbabwe reporting more years of experience with providing contraceptive implants to women and greater numbers of insertions and removals performed.

**Table 2:** Demographic characteristics of in-depth interview participants experienced in implant insertion and/or removal.

	South Africa (SRC) (n = 8)	Zimbabwe (PZAT) (n = 9)	Total (n = 17)
**Age mean (median, min-max)**	49.5 (46.5, 33–73)	39.7 (38, 30–53)	44.3 (40, 30–73)
**Female/Male**	5 (63%) / 3 (38%)	5 (56%) / 4 (44%)	10 (59%) / 7 (41%)
**Years of experience as contraceptive implant provider, mean (median, min-max)**	3.25 (3, 2–5)	8.1 (8, 3–13)	5.8 (5, 2–13)
**Approximate number of implant insertions performed**			
0 insertions	3 (38%)	0 (0%)	3 (18%)
1–100 insertions	3 (38%)	3 (33%)	6 (35%)
101–1000 insertions	2 (25%)	4 (44%)	6 (35%)
>1001 insertions	0 (0%)	2 (22%)	2 (12%)
**Approximate number of implant removals performed**			
1–100 removals	7 (88%)	4 (44%)	11 (65%)
101–1000 removals	1 (13%)	3 (33%)	4 (24%)
>1001 removals	0 (0%)	2 (22%)	2 (12%)

*Note*: SRC, Setshaba Research Centre; PZAT, Pangaea Zimbabwe AIDS Trust.

### Implant preferences

Women participants and health care providers with experience administering contraceptive implants agreed in multiple ways about the preferred characteristics of an MPT implant for contraception and HIV prevention, although there were some notable differences in their preferences and considerations. The results presented in this analysis focus on three key areas that participants identified as facilitating rollout, uptake, and adherence to a MPT implant: preferences around discreetness, independent retrievability for each indication, and key factors that would facilitate or impede social adoption. Illustrative quotes that align with these key areas are shown in Table 3.

**Table 3:** Illustrative quotes shown by key results theme.

	Implant experienced	Implant naïve	Health care providers
Implant preferences related to discreetness, physical location, and flexibility	*It [discreetness] is important because if my partner engages sexual activities with other women*, *as long as you are protected—including my unborn baby—with the implant*, *I am fine with it*. *So*, *it’s more of a good secret to keep for us as women*. (Zimbabwe, post-partum)	*I don’t want it to be visible*. *My boyfriend can take advantage and say*, *is it you are using a family planning method*. *So*, *they start doing what they want*. *So*, *if it’s not visible its ok*. *It will be my secret*. (Zimbabwe, engaged in transactional sex)	*So as a person who is doing insertions and removal*, *I would prefer something that is easily palpable*. *But I have noticed that it is a concern with some women if it is easily palpable*, *probably because they don’t want other people to know that they have something under their skin*. (Zimbabwe, medical doctor and researcher)
	*I don’t care about people*. *It just about me feeling it*. (South Africa, engaged in transactional sex)	*I want it to go like this [feels smoothly]*, *it shouldn’t show*. *I only want that little dot [scar] to be visible*. *But when it is stiff*, *I am going to keep on feeling it*, *be like ‘oh I have inserted it’*… *When a person touches me*, *they will be like ‘what’s going on’*? (South Africa, nulliparous)	*People need to feel*, *see*, *for them to be reassured*. *If it’s not easy to feel they might feel like they don’t have control*. *They don’t know whether they still protected or not*. *So when they can feel something*… *they feel like they have some control*. *Okay it’s still there*, *I’m still protected*. *So I think the firmer one is better*. (South Africa, medical doctor)
	*Something that you don’t feel*, *I will end up thinking that is it still serving its purpose*? *Is it still there*? *So*, *I’ll end up questioning myself if I don’t feel it*, *but if I feel it am happy with it*. (Zimbabwe, post-partum)	*But you know when it comes to one’s life and safety*, *it doesn’t matter whether it is visible or not*. *It will be fine… As long as I am safe*, *so who says what*, *I don’t care*. (South Africa, nulliparous)	*I would still prefer the arm*. *It’s easy*, *it gives an easy position to insert*. *It will be easy to observe as you go on and it’s not actually visible to other people*. *It can still be private*. *You still have it on your arm but nobody can see it*. *Then you can view it with ease*, *you can palpate it with ease*. (South Africa, nurse)
Implant preferences related to discreetness and biodegradability	*I think that*, *it’s ok not to have it removed because if I think of the removal*, *I think of the pain*. *And that pain is what sometimes makes a woman avoid going for removal*. (Zimbabwe, engaged in transactional sex)	…*It dissolves and goes where*?! *I want to know where does it go*… *It just disappears [facilitator laughing] in my skin*? *[Facilitator and participants laughing]*. *Where does it go*? (South Africa, engaged in transactional sex)	*From a provider’s point of view I prefer the dissolvable one*. *It means less work and no removals*. *It’s cost effective to the clients*. *But the client might not feel good*. *As they will be asking themselves that is it truly gone*? *Some prefer having it removed and seeing it*. (Zimbabwe, medical doctor, researcher, and trainer)
	*I prefer the one that dissolves because I once got a C-section and stitches dissolve but I haven’t heard any side effects*, *unlike being cut open every now and again when they want to remove it*. *I think it’s not necessary because some of these things have to happen naturally*. (Zimbabwe, post-partum)	What do you think about an implant that dissolves in your body and you wouldn’t need to be removed? *P1*: *I think it is fine because there is no labour of cutting the same position it was inserted in the process leaving another scar*. *P2*: *I don’t like it*. *There are too many side-effects affecting people*. *It might dissolve and affect you*. *You might get cancer*, *skin disease or other diseases*. *So*, *I don’t like it*. *P3*: *I think it causes too many problems*. *If it dissolves it may cause cancer or other diseases*, *hypertension*, *diabetes might be triggered*. *P4*: *I didn’t see any problem because you said it’ll pass out of the body as one passes out waste*. *So how will it affect someone yet it is flushed out of the body*? (Zimbabwe, post-partum)	*I’ll be delighted to have it because we are causing trauma to the patients by removing it*, *inserting it again*, *removing it so it’s more traumatic for the patient*. *But the questions that I think the patients will ask is it not going to cause cancer because it dissolves in my body*? *Is it not going to—am I going to fall pregnant again and is it going to protect… how long is it going to protect me from HIV and*, *and pregnancy if it disappears*. *So I think those are the questions that might be asked by the patients*. (South Africa, nurse and clinical officer)
	*I would choose the one that melts*… *Implanon they have to take it out again and when they take it out*, *they stitch you*. (South Africa, engaged in transactional sex)	*I think the one that dissolves is good*. *It will be less painful because you don’t have to go back to have them [health care providers] take it out*, *things like that*. *So when it dissolves by itself and you only have to go insert another one I think it is convenient*. (South Africa, post-partum)	*It’s easy to insert the implant and it usually leaves a small minimal scar*. *You know*, *but now if the implant it’s too deep and then it’s—you struggle to remove it*, *you might find yourself having made a bigger scar just trying to remove… Yeah so if it’s something that will be easily inserted with a minimal scar and that could dissolve in the body that would be beautiful*. (South Africa, medical doctor)
Implant preferences related to independent retrievability	*…If ever I meet Mr*. *Right*, *I will be able to remove this one [contraceptive component] so that I can have a child*… *And the one for diseases I actually don’t want it to be removed*. (South Africa, engaged in transactional sex)	*I think that it is important to remove one and remain with the other*. *Because after getting married*, *and you are now living with him*, *how would you handle it when you can’t get pregnant*? *If you remove the pregnancy one and remain with the HIV one*, *then at least you will be home and dry [assured]*. (Zimbabwe, nulliparous)	*Now I think it [separate rods] will be a good thing*. *Because at least the woman will be still protected*. *You know the most important part is to help our patient not to contract HIV*. *You know*. *Yes so if our patient now wants to have a baby and maybe the husband or the boyfriend is HIV positive*. *At least it’s going to be safer*. (South Africa, nurse)
	*I personally would be happy about it [independent retrievability] because I may want to have a child but I don’t want to contract HIV*. *So*, *if I am able to do that I don’t see any problem*. *Don’t know about others*. (Zimbabwe, post-partum)	*I think that before you insert it*, *you must think it through*. *It shouldn’t be the case of you removing one and leaving the other*. *Like have a one-on-one meeting with yourself*. (South Africa, nulliparous)	*I would go for one that has two rods; one for HIV and one for contraception mostly because of the change in contraception or HIV prevention needs over time… The only challenge will then be if the two rods are the same in terms of length*, *thickness and flexibility*. *The challenge would then be in identifying which one is which if you are to remove one and to keep the other*. (Zimbabwe, medical doctor and researcher)
Social adoption factors	So how would you want to learn about it? *P2*: *TV*, *radio*, *billboards*, *peers*, *any mode of communication because we want it to reach everyone*. Ok, anyone else? *P1*: *I think from all modes of communication because if people keep on talking about things*, *it raises an awareness*… (Zimbabwe, nulliparous)	*P1*: *It’s not good for them to be asking me what I am using and why I am using it*. *They should stick to their work and give what I came for so that I leave*. [Laughter]… *P2*: *Yes*. *I wanted to say the same thing that anywhere is ok but it’s the attitude of the workers*. *They should have good hearts*. (Zimbabwe, nulliparous)	*Since most women are going to go to a clinic for family planning services*, *it will be within the family planning department of a clinic*. *The way that most of the poly clinics*… *and as far as clinics are arranged is such that you have got your antenatal care*, *your baby clinics*, *family planning services*, *HIV testing*, *usually around the same area*. (Zimbabwe, medical doctor and researcher)
	*They should form aah*, *aah groups that are going to do [go] door-to-door*… *Cause there are some people who don’t like walking [being outdoors]*… *At least they must—Where they know that that community really is involved in*, *in sex*, *too much [a lot]*. *They are exposed to HIV too much*. (South Africa, post-partum)	…*There is a possibility of there being mistakes made by the person who is inserting it*. *Cause right now the nurses*, *the nurses at the clinics they are rough*, *they don’t want to work [do their job]*. *So you find that even though they have inserted one [implant]*, *they end up being rough [with you]*. *So no*, *I think they would make mistakes*. (South Africa, nulliparous)	*Yeah*. *Uhm*, *like with us we’ve got the youth friendly clinic somewhere far away from our clinic*. *It’s helping because most of the young ones are going there*, *the people who wants to do prevention*. *They go there and it will be easier because the sister won’t be doing other things*… *she will be concentrating on it*. (South Africa, nurse and clinical officer)
	Where would you want to access the product? *P1*: *Family Planning clinics because it “includes” women only*. Ok. Don’t women also go to OI [Opportunistic Infection] clinics? *P2*: *They do but then people will start talking about us if they see us at the OI clinic*. *Iii*, *people will be pointing at us saying*, *“Eh*, *did you see her*?*” [Some participants laugh and voice their agreement*.*]* (Zimbabwe, engaged in transactional sex)	…*At the clinics*, *the nurses are rough*. *And they don’t care that*, *like they just do it because you came to the clinic*, *they’ll just help you cause they have to help you*. *So you find that maybe they will be inserting it*, *she is just inserting it because just [she has to]*. *She doesn’t check whether she is inserting it correctly*. (South Africa, nulliparous)	*First people want to know about it*. *So you should do road shows*, *they are one of the most powerful advertising tool*. *Then mass media during the main news*, *it will capture everyone’s attention is*. *Even during the prime time and explain the new method*. *Also use those village health workers; they reach those hard to reach areas*. *So you can use them once you equip them with knowledge*. (Zimbabwe, nurse)
	*…You might go to the hospital and find those old nurses who are not motivated*. *Would they insert all the rods properly [laughter breaks]*? *Ladies let’s be honest with each other*. (Zimbabwe, nulliparous)	*Those mobile clinics attract everyone in the community and once you do it there*, *the community judge you but at the clinic it’s a bit private*. (Zimbabwe, implant naïve, post-partum)	*Education*. *Education*. *Health education by the nurses*. *Education at radios*. *The pamphlets*. *I think if we give them information*, *enough information they will come for it*. (South Africa, nurse and clinical officer)

### Preferences relating to implant discreetness

The importance of discreet implant use was the most salient topic that emerged among women and health care providers. Discussions about implant flexibility/palpability, subcutaneous location of implant on the body, and the potential for a biodegradable implant revealed a tension between two key attributes: the ability to use the implant discreetly as compared with having a physical indicator that the implant is present and effectively releasing drugs. Perspectives on how to balance these divergent requirements, for participants, were shaped by the level of experience with the contraceptive implant, influence of sexual partners, and experience with transactional sex. For health care providers, perspectives were shaped by experience with insertions and removals of the contraceptive implant, views expressed by clients, and ease/comfort of prior implant insertions and removals.

#### Discreetness, physical location, and implant flexibility

Among focus group discussion participants, preferences for discreet use directly informed perspectives around bodily location of the implant placement and rod flexibility. The desire for a more flexible implant and a less noticeable insertion location was driven by an interest in invisibility. This invisibility was associated with avoidance of conflict in sexual relationships, families, friendships, and communities. Although the greatest proportion of women preferred the familiar placement site on the upper arm, others expressed interest in the thigh, buttocks, and side (Table 4). Many women expressed the need to hide the use of the implant from a husband/sexual partner because some men do not want them to be on contraception or would not support the use of an HIV prevention product. For women who felt their partner would accept their use of contraception, the conventional arm location was regarded as appropriate for the SCHIELD implant because partners are familiar with this location and would not suspect the use of an MPT. For example, one participant stated, *“So even if the husband holds your hand and sees it (SCHIELD implant)*, *he’ll just think it’s Jadelle*, *though I’ll be knowing that I removed it way back*. *It’ll be my secret”* (Zimbabwe, postpartum, implant experienced). Women who said a partner would disapprove of contraception use preferred a different location. A few recommended the thigh as a discreet location; however, some participants with transactional sex experience said that this is a location that a man would be more likely to touch the skin and were concerned he would feel the implant.

**Table 4:** Anatomical site preference data collected through votes in focus group discussions and through the quantitative questionnaire after completing in-depth interviews.

Body Location Preference	South Africa (SRC)	Zimbabwe (PZAT)	Total
Focus Group Discussions	**N = 46**	**N = 64**	**N = 110**
Arm	20 (43%)	27 (42%)	47 (43%)
Thigh	11 (24%)	16 (25%)	27 (25%)
Buttocks	8 (17%)	7 (11%)	15 (14%)
Side	7 (15%)	14 (22%)	21 (19%)
In-depth Interviews	**N = 8**	**N = 9**	**n = 17**
Upper arm	3 (38%)	9 (100%)	12 (71%)
Upper thigh	1 (13%)		1 (6%)
Outside thigh	1 (13%)		1 (6%)
Upper buttocks	2 (25%)		2 (12%)
Other (belly)	1 (13%)		(6%)

*Note*: SRC, Setshaba Research Centre; PZAT, Pangaea Zimbabwe AIDS Trust.

Among implant-experienced and implant-naïve participants there were nuanced differences in the descriptions of why body location was important, especially as it related to discreetness (or lack thereof). Among women who were implant experienced, a minority explained that they would want to palpate the implant to make sure it is still present and in place. The arm was seen as the easiest location for monitoring, as compared with other parts of the body such as the thigh or buttocks. When implant-naïve participants spoke about the need for discreet implant use, they were more likely to emphasize concerns about issues with people in their lives, particularly partners, but also friends and relatives. One participant explained that if her boyfriend knows that she is using an MPT, he will take advantage of that and see other partners. Others mentioned that they do not want people to know that they are using a contraceptive or HIV prevention method because it will reveal the participant is sexually active and may lead to sexual stigma and connotations with promiscuity. The arm was preferred by some of these women because it is an area that a sexual partner is less likely to touch and can easily be covered by clothing. Others disliked the arm because men know to look there if a woman has an implant, and consequently they would prefer a different insertion site.

Flexibility of the rods also was seen as an important factor when discussing discreetness or the desire to be able to monitor an implant’s location. While some women preferred an implant as stiff as the Implanon implant, others preferred an implant as flexible as the Jadelle implant or wanted it to be even more flexible than Jadelle. A dominant theme was that a flexible implant would be less visible under the skin and less likely to be seen by partners who may disapprove use of prevention products. Implant- experienced women were more likely to prefer a palpable implant to feel if it remains in place or if it has started dissolving through the biodegradation process.

Health care providers expressed differing views about the level of stiffness/flexibility of the rods. Most providers stated that the SCHIELD implant should be on the firmer side because they believed that it needs to be firm enough for women to feel it, reassuring them that it is there. One doctor stated, *“It has to be palpable*, *especially to the client*. *The more they feel it; it gives them a sense of satisfaction*. *To the service provider*, *they will prefer a palpable one just in case one wants to remove it*. *Palpable ones are easier to remove than nonpalpable ones”* (Zimbabwe, medical doctor). Health care providers also stated that firmer implants will increase ease of insertion and removal. They illustrated this by bending the Jadelle and Implanon. They stated that if the SCHIELD implant were more flexible than Jadelle, the risk of the implant losing its structural integrity during removal would be higher. A minority view among health care providers was that the SCHIELD implant should be more flexible, similar to Jadelle, because it would be important for women who do not want to disclose use to partners/other people in their lives. One provider explained, *“Most women*, *they go into prevent without informing the partner*. *That you know what*, *I’m on a method of preventing*, *I don’t want to have kids*. *So*, *if it’s somewhere that can be easily palpable it might raise questions”* (South Africa, public clinic nurse).

When considering where they thought would be the best location for patients to have an MPT implant inserted, health care providers preferred the upper arm. However, one provider stated that scarring on the arm would be more visible than the thigh, indicating that the thigh could be a more discreet location for insertion. Providers who preferred insertion in the arm stated that women are already accustomed to this location, although they recognized that the thigh and belly could be seen as discreet.

#### Impact of biodegradability on discreet use

Most women and health care providers at both sites preferred a biodegradable implant (Table 5). Women’s opinions about biodegradability reflected the competing desire for discreet use and the interest in being able to monitor use. A major advantage of a dissolving implant was described as the reduction in scarring caused during implant removal, although there was debate over the impact of scarring from insertion and removal of the implant. Some participants said protection was more important than concerns about scarring, whereas others said that noticeable scars can make it difficult for them to use the implant because they live with family or a partner who might question them about the scarring. Religious groups and the community at large also were cited as social groups who may disapprove of implant use, thus increasing the need to conceal use.

**Table 5:** Participant preferences for dissolvability.

Do you prefer a dissolving implant (doesn’t need removal) or a non-dissolving implant (must be removed?)	South Africa (SRC)	Zimbabwe (PZAT)	Total
Focus Group Discussions	N = 46	N = 64	N = 110
Dissolving implant	40 (87%)	59 (77%)	89 (81%)
Nondissolving implant (needs removal)	6 (13%)	15 (23%)	21 (19%)
In-depth Interviews	**N = 8**	**N = 9**	**n = 17**
Dissolving implant	8 (100%)	6 (67%)	14 (82%)
Non-dissolving implant (needs removal)	0 (0%)	2 (22%)	2 (12%)
Doesn’t matter/no preference	0 (0%)	1 (11%)	(6%)

*Note*: SRC, Setshaba Research Centre; PZAT, Pangaea Zimbabwe AIDS Trust.

Primary motivations for wanting a biodegradable implant aligned between the implant-experienced and implant-naïve groups. First and foremost, views were shaped by the attractive feature of avoiding pain associated with implant removal. Implant-experienced women highlighted the reduced need for clinic visits as shaping their interest in a biodegradable implant. Discussing the benefits of an implant that dissolves, one explained, *“Because when they remove it*, *it is painful*. *So*, *I think that it is fine*, *it can just dissolve on its own”* (South Africa, nulliparous, implant experienced).

Concerns regarding the biodegradable implant, however, varied between these two groups. Although a concern was voiced across focus group discussions about the fate of the implant after biodegradation, this was particularly pronounced among implant-naïve women. Several implant-naïve women expressed anxieties around an implant that dissolves in the body, as one stated: “*I want clarity about where it goes before I agree that it should dissolve inside my body”* (South Africa, implant naïve, engaged in transactional sex). Some wanted to know when it would start dissolving, what happens to the dissolved particles of the implant, and at what point in the dissolving process would a new implant be inserted. One participant said she prefers a nondissolving implant because it makes her feel confident that the implant is still in place and offering protection. Other women were more concerned about the potential side effects or diseases like cancer that could result from a dissolving implant. Some participants expressed that their top priority was an implant that is effective and they were less concerned about it being biodegradable or not.

Most health care providers concurred with the women’s views and, in their capacity as providers of implants, preferred the dissolving implant (Table 5). They were confident that end users would accept an implant like SCHIELD given that women described how traumatic the removal process can be. Health care providers thought that women’s past experiences with removals would likely make them opt for the dissolving implant. A few providers discussed how scarring from the insertion/removal can make it difficult for women to use the implant discreetly. One felt that a biodegradable implant would help with reduced scarring. Two providers explained that an issue with the insertion in the arm was that some nurses tend to make big lacerations during removal, which does not look nice cosmetically because the scars are pronounced.

Health care providers concurred that the biodegradable implant would help reduce hospital visits because there is no need for removal, which also will reduce the cost of the implant as the number of removals is reduced. One provider stated, *“I think it’s good as it saves time for both the client and health care workers”* (Zimbabwe, nurse). Another provider raised questions about when the implant would start to dissolve and the duration of use. Providers from both sites stated that many women are familiar with dissolving stitches, which would help with implant acceptability. However, some health care providers talked about the need for proper counseling because an appropriate explanation of the concept of a biodegradable implant may increase acceptability and help in dispelling myths.

### Independent retrievability

Participants were asked about their preferences for an MPT that is coformulated in one rod as compared with an MPT that is coadministered as two separate rods. Many women were in support of the idea of independently retrievable rods (one rod for HIV prevention and one rod for contraception) because it would allow them to consider taking out the contraceptive portion earlier. Quantitative data collected after the focus group discussions confirmed that 61% of women preferred independent retrievability overall, although there was a difference across sites: 70% of women in Chitungwiza/Harare preferred independently retrievable rods, compared with 48% of women in Soshanguve (Table 6). Women who preferred independent retrievability noted that it provided the possibility of allowing them to adapt their prevention product as their life circumstances changed. This rationale was echoed across participant groups and sites.

**Table 6:** Preference among participant types for independent retrievability (rather than a combined formulation) for the HIV and pregnancy prevention indications.

Preference for Independent Retrievability	South Africa (SRC) n/N (%)	Zimbabwe (PZAT) n/N (%)	Total n/N (%)
Focus Group Discussion Participants	22/45 (48%)	45/64 (70%)	67/110 (61%)
**Nulliparous**	7/14 (50%)	16/26 (62%)	23/40 (58%)
**Post-partum**	9/18 (50%)	13/18 (72%)	22/36 (61%)
**Engaged in transactional sex**	6/14 (43%)	16/20 (80%)	22/34 (65%)
In-depth Interviews	5/8 (63%)	7/9 (78%)	12/17 (71%)

*Note*: SRC, Setshaba Research Centre; PZAT, Pangaea Zimbabwe AIDS Trust.

Preference for independent retrievability was also examined by contraceptive implant experience status at each site. While there was no significant difference in preference between the implant-experienced and implant-naïve women in South Africa (47% as compared with 48%, respectively, p = 1.00), a difference was seen between the groups in Zimbabwe, with implant-experienced participants more likely to prefer independently retrievable implants when compared with participants who had not used a contraceptive implant previously (88% as compared with 60% respectively, p = 0.025).

A discussion about changing fertility intentions was common among women at both sites, although it was discussed frequently within the context of getting married at Chitungwiza/Harare (this was less pronounced at Soshanguve). In addition to a desire to conceive, potential end users discussed what else might cause them to remove one or both indications of an MPT implant early. Reasons for removal included pressure from male partners or parents; impact on menstrual cycle; and side effects such as headaches, vomiting, and loss of appetite. As the participants debated the value of independent retrievability of the hypothetical implant, they expressed concerns about how competent the health care providers would be at the removal procedure.

Most health care providers agreed with the women, indicating they preferred the option of having the HIV prevention and contraceptive portions of the implant separated into independently retrievable rods. Providers who supported this idea spoke about the convenience of removing the unwanted portion. The one provider who preferred a combined implant felt like it would be “simpler,” indicating that the idea of having the option to have one or the other rod removed early might confuse clients. Providers spoke about a variety of ways the two rods could be distinguishable after insertion to aid in cases of independent removal. Some providers thought inserting rods in separate sites would be the easiest way to facilitate early removals from a provider/logistics/efficiency perspective, although two of providers recognized the drawback to the patient because of the pain of insertion and issues of having two scars. Differing rod lengths (i.e., a shorter rod for a contraceptive component and a longer rod for an antiretroviral component) were discussed, but often the shorter rod (10mm length) was anticipated to be difficult to locate for early removal, if needed.

### Social adoption factors

Women raised concerns about health care providers who were perceived as unmotivated and hypercritical toward young women who use prevention products. They expressed that if stigma and judgement are not addressed, it may affect uptake of the 2-in-1 implant, particularly among young unmarried women. These participants further suggested training for health care providers to improve bedside manners and service provision to younger women seeking contraceptive and HIV prevention options. The women also said that health care providers in general, not just those dealing with sexual reproductive health issues, should be given adequate information concerning a future MPT implant.

Women offered several suggestions around combating stigma and misconceptions around the implant. These included awareness campaigns in the communities on existing MPTs (including SCHIELD, when available) and involving male partners during counseling sessions. While some potential end users said their partners have no problem with them using contraceptive implants, they anticipated challenges if they revealed that they are using a 2-in-1 implant with an HIV prevention component.

Similarly, health care providers placed a strong emphasis on training so that they can provide comprehensive counseling on MPT implants. Both pre- and post-insertion counseling were mentioned as important because they help women to make informed decisions and address their questions and concerns. Some providers felt that the concept of the dissolving implant may be challenging to comprehend, requiring providers to be competent to explain it. Providers believed comprehensive counseling would help increase uptake of the product if it addressed issues such as potential side effects, biodegradability, duration of use, scarring, and independent retrievability.

While health care providers held varying opinions on implant access, a majority agreed that women would most likely want to access the implant from clinics because clinics already offer family planning and HIV prevention services. Some providers favored family planning clinics because SCHIELD would have a contraceptive component and that there was little stigma when being attended to at such clinics. Other providers thought that SCHIELD could be accessed from HIV clinics, but several felt potential end users may fear the stigma associated with seeking services from HIV clinics. Youth-friendly centers also were mentioned as ideal locations for young women to access an MPT implant because they would be comfortable getting services there. Mobile clinics were mentioned to reach locations where women have difficulty accessing a clinic. However, providers questioned the comprehensiveness of the counseling offered in mobile clinics as they felt these clinics are more concerned about the targets achieved as compared with the quality of service provided.

Health care providers agreed that the community should access information about an MPT implant from various sources. Providers in facilities who are trained and competent to offer the product would be a key source of information. One provider emphasized that women would feel comfortable hearing about such products from providers from within their communities, *“I think it is the face that you are put in front that is more important”* (Zimbabwe, medical doctor).

Both the women and health care providers thought that SCHIELD should be included in the health education that is given at the facilities and it should be backed by educational and communication materials such as pamphlets and fliers. Although all participants discussed the pros and cons of whether the product was seen as a family planning or an HIV prevention product, ultimately 75% of women and 94% of providers thought that a 2-in-1 implant should equally emphasize the pregnancy and HIV prevention indications in its packaging and advertising, rather than only one or the other **(Table 7)**. Among the focus group discussion participants who did not prefer a 2-in-1 framing, almost all preferred an HIV prevention emphasis. Focus group participants and providers suggested that information about SCHIELD could be disseminated through other forms of media, such as television, radio, and newspapers. Some suggested generating community awareness by making use of community-based organizations that could talk about the SCHIELD implant at social clubs, churches, and schools. Word of mouth also was recommended as a strategy in which early adopters could act as peer educators and spread the word about the implant.

**Table 7:** Packaging and advertising of an MPT implant.

How Should an MPT Implant Be Advertised and Packaged?	South Africa (SRC)	Zimbabwe (PZAT)	Total
**Focus Group Discussions**	**N = 46**	**N = 64**	**N = 110**
HIV prevention (+ family planning)	13 (28%)	11 (17%)	24 (22%)
Family planning (+ HIV prevention)	2 (4%)	1 (2%)	3 (3%)
2-in-1 (equal emphasis)	31 (67%)	52 (81%)	83 (75%)
**In-depth Interviews**	**N = 8**	**N = 9**	**n = 17**
HIV prevention (+ family planning)	0 (0%)	0 (0%)	0 (0%)
Family planning (+ HIV prevention)	1 (13%)	0 (0%)	1 (6%)
2-in-1 (equal emphasis)	7 (88%)	9 (100%)	(94%)

*Note*: SRC, Setshaba Research Centre; PZAT, Pangaea Zimbabwe AIDS Trust.

## Discussion

To our knowledge, this is the first study examining the views of potential end users and health care providers on an MPT implant for HIV and pregnancy prevention in preclinical development. During qualitative interviews and focus group discussions, participants saw and handled different implant prototypes and offered insight into implant design factors and contextual issues they perceived would influence successful rollout of an MPT implant. Participants expressed enthusiasm for an MPT implant and identified three key areas for consideration during product development: discreetness, ability to independently retrieve the HIV or pregnancy prevention component, and social adoption considerations. Differences emerged in the qualitative analysis between the implant-naïve and implant-experienced user groups for some preferences, such as palpability and biodegradability. However, it is notable that key differences did not emerge between participants who were recruited because they were nulliparous, post-partum, or engaged in transactional sex work.

The study findings regarding the importance of discreetness among both providers and women end users align with previous studies showing the importance of “invisibility” and discreet use of other HIV [16, 22], pregnancy prevention [23, 24], and MPT products [8, 25–31] across a variety of form factors. Having the option to use a product discreetly has been identified as a key driver of MPT product acceptability, particularly as it relates to negotiation, secrecy, or disclosure to loved ones and male partners [8, 32–34]. Oral PrEP users and participants in studies of oral MPT products express concerns about lack of privacy to take and store pills leading to inadvertent disclosure and ensuing stigma related to PrEP use or assumptions of HIV infection [28, 30, 34–37]. While many end users highlight the ability to use vaginal rings discreetly as a positive attribute [28, 33, 38], others express hesitations about the ring being detected by partners during sex [28, 34, 39, 40]. While contraceptive injections avoid many of these challenges and are, indeed, widely used in sub-Saharan Africa, the first-generation injectable option (CAB-LA) requires reinjection every two months, is painful, and necessitates frequent clinic visits, a potential access barrier [3]. For implants specifically, discretion is a unique issue, as it can be visible and palpable under the skin, yet it does not require home storage, frequent clinic visits, or the more visible action of swallowing a pill or putting on a condom. In the case of the SCHIELD implant, there are opportunities to make design choices that would enhance discreetness (such as more flexible or smaller rods, or insertion in different body locations) to address these concerns.

An implant design that would allow for independent retrievability of the HIV and pregnancy prevention indications as life circumstances may change was desirable among most women and health care providers in this study. This is a unique feature of an implant delivery system, as other MPTs in the pipeline such as vaginal rings, inserts, and gels would not easily facilitate continuation of one indication while removing the other. Although this is an attractive feature, it presents technical challenges around identification and removal of a single component, appropriate tracking of which indications were currently protected, and whether independent removal may lead to additional scarring. Grappling with the implications of this potential benefit now may help product developers decide how this feature should be prioritized as they move through the product development process. It is important to note that the key findings highlighted relating to discreetness, flexibility, biodegradability, and social adoption largely reflect broader concerns around social issues such as gender norms, stigma, misinformation, and relationship and power dynamics with male partners, rather than concerns about the actual features of the product itself. This emphasizes the need to address contextual issues to reduce uptake barriers and persistence for any novel MPT, especially those that are administered by health care providers.

Male partners were identified as key influencers concerning future adoption of an MPT implant. Male partners have been identified as important to women’s MPT acceptability through indirect influence on women’s perceptions of MPT product attributes and direct influence on women’s decision-making [8, 33, 34, 37, 41–44]. Other social actors such as religious communities, parents, and health care providers also were seen as important actors in future rollout efforts. Although these people could be important facilitators to uptake if they are supportive and well-informed, participants in this study thought they also could be detrimental if not properly sensitized. This fed into strong opinions around features such as biodegradability and discreetness, insofar as they would allow a woman to use an MPT implant despite potential opposition from community members. There has been limited research on how community-related factors might impact MPT acceptability and uptake, though concerns around stigma associated with using an MPT product due to assumptions around sexual behavior or HIV status have been noted [8, 30, 45–48]. While health care providers engaging in research related to MPTs under development have generally been supportive of the health benefits and clinic efficiencies that could be achieved with MPTs [48, 49], some end-users have expressed concerns about providers’ stigmatizing attitudes toward young women and married people using MPTs [33, 48, 50].

### Limitations

This was primarily a qualitative study recruited through purposive sampling of specific subgroups of interest. Additionally, all participants were recruited from geographically specific areas in Soshanguve, South Africa, and Chitungwiza and Harare, Zimbabwe. Consequently, the generalizability of these findings is limited. This approach, however, allowed elicitation of insights from specific groups of high interest for future MPT implant rollout and generated relevant data to inform product development, including implant size, duration, number of rods, flexibility, and biodegradation. As in other similar studies, there was a risk of social desirability bias, and participants may have felt a need to express positive views around the MPT implant being discussed. It is reassuring to note that participants did describe concerns about the SCHIELD implant. Finally, the findings around social factors for adoption are not easily modifiable as they represent larger contextual and structural issues. Some aspects of the implant design—such as size, location, flexibility, and biodegradability—could mitigate the potential social factors, and counseling messages and community sensitization may mitigate these broader issues.

## Conclusions

The findings from health care providers and women end users in two countries provided key areas of consideration for the design of an MPT implant for HIV and pregnancy prevention. Given the differing preferences among women end users, the unique views of health care providers, and the limitations of product design changes for an MPT implant, future research that requires women to prioritize the most important features of an MPT implant will be critical. The results are forthcoming from a large, quantitative follow-up study to define the most important attributes systematically and robustly in PrEP and MPT implants. Strategies that allow for new technologies to be better tailored to end users during early product development stages, through the inclusion of end user and other key stakeholder voices, may have a greater likelihood of being adopted and used in the real world.

## Supporting information

S1 Data (XLSX)
